# PEAK1 Acts as a Molecular Switch to Regulate Context-Dependent TGFβ Responses in Breast Cancer

**DOI:** 10.1371/journal.pone.0135748

**Published:** 2015-08-12

**Authors:** Megan Agajanian, Anaamika Campeau, Malachia Hoover, Alexander Hou, Daniel Brambilla, Sa La Kim, Richard L. Klemke, Jonathan A. Kelber

**Affiliations:** 1 Department of Biology, California State University Northridge, Northridge, CA 91330, United States of America; 2 Department of Biology, Georgetown University, Washington, DC 20057, United States of America; 3 Department of Pathology & Moores Cancer Center, University of California San Diego, La Jolla, CA 92093, United States of America; Wayne State University School of Medicine, UNITED STATES

## Abstract

Transforming Growth Factor β (TGFβ) has dual functions as both a tumor suppressor and a promoter of cancer progression within the tumor microenvironment, but the molecular mechanisms by which TGFβ signaling switches between these outcomes and the contexts in which this switch occurs remain to be fully elucidated. We previously identified PEAK1 as a new non-receptor tyrosine kinase that associates with the cytoskeleton, and facilitates signaling of HER2/Src complexes. We also showed PEAK1 functions downstream of KRas to promote tumor growth, metastasis and therapy resistance using preclinical *in vivo* models of human tumor progression. In the current study, we analyzed PEAK1 expression in human breast cancer samples and found PEAK1 levels correlate with mesenchymal gene expression, poor cellular differentiation and disease relapse. At the cellular level, we also observed that PEAK1 expression was highest in mesenchymal breast cancer cells, correlated with migration potential and increased in response to TGFβ-induced epithelial-mesenchymal transition (EMT). Thus, we sought to evaluate the role of PEAK1 in the switching of TGFβ from a tumor suppressing to tumor promoting factor. Notably, we discovered that high PEAK1 expression causes TGFβ to lose its anti-proliferative effects, and potentiates TGFβ-induced proliferation, EMT, cell migration and tumor metastasis in a fibronectin-dependent fashion. In the presence of fibronectin, PEAK1 caused a switching of TGFβ signaling from its canonical Smad2/3 pathway to non-canonical Src and MAPK signaling. This report is the first to provide evidence that PEAK1 mediates signaling cross talk between TGFβ receptors and integrin/Src/MAPK pathways and that PEAK1 is an important molecular regulator of TGFβ-induced tumor progression and metastasis in breast cancer. Finally, PEAK1 overexpression/upregulation cooperates with TGFβ to reduce breast cancer sensitivity to Src kinase inhibition. These findings provide a rational basis to develop therapeutic agents to target PEAK1 expression/function or upstream/downstream pathways to abrogate breast cancer progression.

## Introduction

Breast cancer is the most common cancer among women, accounting for 23% of all cancer cases [1]. Patients with metastatic forms of this disease have a 24% survival rate [2]—thus, understanding the molecular regulation of the metastatic cascade as well as the growth of metastatic tumors can illuminate novel strategies for increasing patient survival.

Transforming growth factor beta (TGFβ) is part of the TGFβ superfamily and acts through the TβRII and TβRI (ALK5) receptor serine/threonine kinases to induce Smad2/3 signaling and gene transcription [3]. In the context of human cancers, TGFβ can act as either a tumor suppressor or a pro-tumorigenic factor capable of inducing epithelial to mesenchymal transition (EMT) and metastasis. EMT is a morphologic and phenotypic shift in cells that is associated with specific changes in gene expression. EMT is essential and strictly regulated during embryogenesis and tissue homeostasis [4]; however, it is deregulated during the progression of epithelial cancers to promote metastasis [5]. During EMT, cells gradually lose their apical-basal polarity, ability to attach to the basement membrane and protein complexes that regulate cell-cell junctions. These changes are also associated with downregulation of epithelial genes (e.g., E-cadherin) and increased expression of mesenchymal genes (e.g., N-cadherin)—the resulting cells tend to migrate more extensively and adopt a more spread, fibroblast-like morphology [4].

As a tumor suppressor, TGFβ exposure promotes cytostasis, apoptosis and differentiation, as well as acting to stimulate a proper immune response [6,7]. However, TGFβ’s signaling mechanisms can be altered to inhibit its anti-proliferative effects and stimulate tumorigenic effects (e.g., EMT) [8]. Interestingly, environmental cues as well as cell type are factors that can determine whether TGFβ acts in a tumor suppressive or tumor promoting manner. While it is understood how the signaling pathways become modified, a complete understanding of the molecular regulation that drives this switch in TGFβ responsiveness remains to be fully elucidated [9,10]. In this regard, TGFβ and ECM/growth factor pathways have been shown to cooperate to promote EMT, migration, invasion and metastasis of breast cancer cells [11,12,13,14,15]. Previous reports have demonstrated that specific extracellular matrix proteins (e.g., fibronectin) can cooperate with TGFβ receptors to shift TGFβ signaling from its canonical Smad2/3 pathway toward non-canonical Src/TβRII/Grb2/MAPK signaling pathways. Notably, this shift has been reported to be a key mechanism through which TGFβ adopts its pro-tumorigenic functions [11,12].

We previously identified PEAK1 (pseudopodium enriched atypical kinase 1, Sgk269) as a novel non-receptor tyrosine kinase that is enriched in the pseudopodia of migrating cells [16,17]. PEAK1 promotes tumor growth/metastasis and therapy resistance in human cancers via its regulation of the actin cytoskeleton and Src, KRas and ErbB2 signaling pathways [16,17,18]. Others have also reported that PEAK1 regulates Shc1 and Grb2 signaling downstream of EGF stimulation [19], and bioinformatics have predicted that PEAK1 may interact with MAPK proteins [17,20]. Finally, PEAK1 overexpression in mammary epithelial cells has been reported to promote an EMT-like response [21].

In this study, we show that PEAK1 can mediate the shift of TGFβ responses from anti-proliferative to pro-tumorigenic in cooperation with ECM-specific signaling events. Using a previously described *in vitro* model for breast cancer progression as well as hormone-responsive breast cancer cells, we show that PEAK1 is necessary and sufficient for TGFβ-induced migration, EMT, metastasis and proliferation in breast cancer. Finally, we demonstrate that this PEAK1-dependent effect occurs via Src/MAPK signaling pathways and that PEAK1 upregulation/overexpression can desensitize breast cancers to the cytotoxic effects of Src kinase inhibition.

## Results

### Increased PEAK1 expression in breast cancer correlates with indicators of poor patient prognosis, mesenchymal gene expression and cell migration

**Fig 1:**
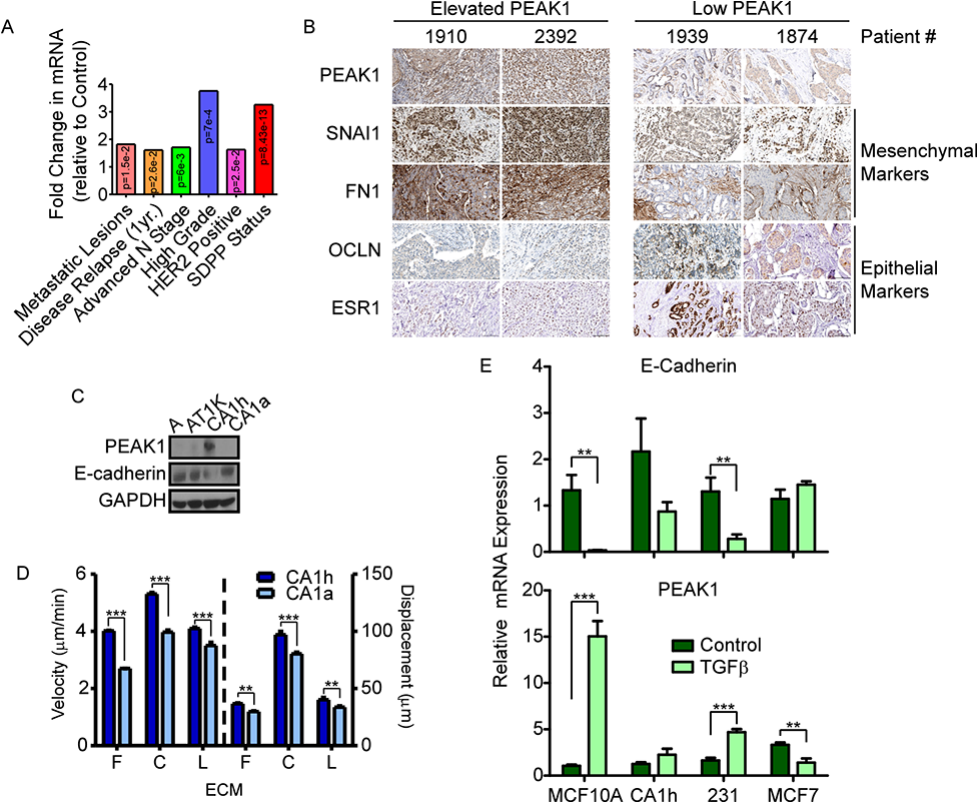
PEAK1 expression in breast cancer correlates with indicators of poor patient prognosis, mesenchymal gene expression, cell migration and is upregulated during TGFβ-induced EMT. (A) PEAK1 mRNA fold change was analyzed from several previous reports of patient data in relation to characteristics that correlate with poor patient prognosis–i.e., metastatic lesions, disease relapse, advanced N stage, high grade, HER2 positive status, and stromal-derived poor prognostic (SDPP) status. (B) IHC from the Human Cancer Atlas of four different patients–two with elevated PEAK1 levels and two with low PEAK1 levels–for SNAI1, FN1, OCLN and ESR1 expression. (C) Western blot analysis on lysates from MCF10A, MCF10AT1K, MCF10CA1h, and MCF10CA1a cells for PEAK1 and E-cadherin expression. (D) Single cell migration assay on 3μg/mL of Fibronectin (F), Collagen (C), or Laminin (L) of CA1h and CA1a cells (velocity is plotted on the left axis and displacement is plotted on the right). (E) MCF10A, CA1h, MDA-MB-231, and MCF7 cells were plated on plastic and treated for 72 hours with TGFβ. RNA was collected and qPCR for E-cadherin (top) and PEAK1 (bottom) expression was performed. ** or *** indicate p-values < 0.01 or 0.001, respectively.

Previous breast cancer microarray studies were analyzed for PEAK1 mRNA expression in relation to markers of poor patient prognosis [22,23,24,25,26]. We found that increased levels of PEAK1 expression correlate with multiple markers of poor patient prognosis, such as metastatic lesions, disease relapse, advanced N stage, tumor grade, HER2 status, and stromal-derived prognostic predictor (SDPP) status (Fig 1A). To evaluate PEAK1 expression in patient samples at the protein level, we selected four breast cancer tissue samples (two with low PEAK1 levels and two with elevated PEAK1 levels) from the Human Cancer Atlas for comparing PEAK1 IHC staining patterns with those of epithelial or mesenchymal markers that have been previously reported to be regulated by PEAK1 overexpression [21,27]. As shown in Fig 1B, samples that have elevated PEAK1 protein levels have increased expression levels of the mesenchymal markers SNAI1 and FN1, while epithelial markers OCLN and ESR1 are low in these samples. The inverse pattern was observed in samples with low PEAK1 protein levels. Analysis of additional markers of EMT [4] revealed that breast cancer samples with high PEAK1 levels expressed reduced levels of the epithelial markers MUC1, E-Cadherin and Entactin and increased levels of the mesenchymal markers Syndecan1 and LEF1 (S1A Fig). We also evaluated the correlation between PEAK1 levels and EMT gene signatures previously reported to be regulated by PEAK1 in mammary epithelial cells [21] across a panel of breast cancer cell lines [28]. Notably, PEAK1 expression correlated with decreased epithelial and/or increased mesenchymal gene expression patterns in HER2+ and/or ER/PR+ subtypes of breast cancer cells (S1B Fig).

Oncogenic HRas transformation of MCF10A mammary epithelial cells and their subsequent *in vivo* selection was the basis for establishing a commonly used model of breast cancer progression [29] and has been shown to represent non-malignant epithelial (MCF10A), breast carcinoma *in situ* (MCF10AT1k), slow-growing breast carcinoma (MCF10CA1h) and fast-growing breast carcinoma (MCF10CA1a) cell types. Upon analysis of PEAK1 and E-Cadherin expression across this panel of four lines, we discovered that PEAK1 levels were highest in the CA1h cells–these also had the lowest E-Cadherin expression levels (Fig 1C) and most mesenchymal morphology of the three HRas transformed lines (S2 Fig). Finally, we found that the PEAK1-high CA1h cells move faster (i.e., velocity) and further (i.e, displacement and track length) than the more epithelial and faster growing CA1a cells (Fig 1D and S3 Fig), irrespective of extracellular matrix substrate protein (i.e., fibronectin, collagen or laminin).

### TGFβ increases PEAK1 expression concurrent with E-Cadherin downregulation

Although PEAK1 has been previously reported to induce EMT-like responses in mammary epithelial cells [21], the contributions of PEAK1 to EMT in breast cancer have not been previously studied. Since TGFβ is a well-known inducer of EMT during both development and cancer progression [21,30], we sought to test the effects of TGFβ on PEAK1 and E-Cadherin expression in MCF10A cells alongside three cellular models of breast cancer. Interestingly, in both the non-tumorigenic MCF10A and highly metastatic MDA-MB-231 cells, TGFβ significantly upregulated PEAK1 and downregulated E-Cadherin (Fig 1E). While there was a trend in PEAK1 upregulation and E-Cadherin downregulation following TGFβ treatment of the CA1h cells, this effect was not statistically significant. Additionally, TGFβ alone was unable to induce EMT or PEAK1 expression in MCF-7 cells (Fig 1E). These data suggest that PEAK1 levels in breast cancer cells are an important factor in determining TGFβ’s ability to induce EMT. In agreement with the possibility that PEAK1 plays a role in EMT and metastasis downstream of TGFβ, we discovered that breast cancer samples from patients with recurrent metastatic disease or poor prognostic biomarker status (i.e., HER2-positive or TNBC) displayed significantly elevated levels of PEAK1 and TGFβ response genes [31,32,33,34] (S4 Fig).

### PEAK1 promotes tumorigenic signaling and proliferation in breast cancer cells

To evaluate the role that PEAK1 plays in TGFβ-induced EMT and metastasis in breast cancer, we proceeded to generate PEAK1-overexpressing MCF7 cells and PEAK1 knockdown MCF10CA1h cells. As shown in S5A Fig (and consistent with our previous studies of PEAK1 in human malignancies [17]), overexpression of PEAK1 increased Erk1/2 and Src pathway activation at either the post-translational modification or protein expression levels. Interestingly, however, we also discovered that PEAK1 overexpression can potentiate Smad2 activation in a TGFβ ligand-independent fashion. When analyzed for their proliferative capacity, PEAK1 overexpression in MCF7 cells led to increases in the number of viable cells as well as S or G2/M phases of the cell cycle (S5B and S5C Fig). Conversely, when PEAK1 was silenced in the CA1h cells, the number of viable cells and the S or G2/M phases of the cell cycle decreased (S5D and S5E Fig).

### PEAK1 potentiates fibronectin/TGFβ-induced EMT

**Fig 2:**
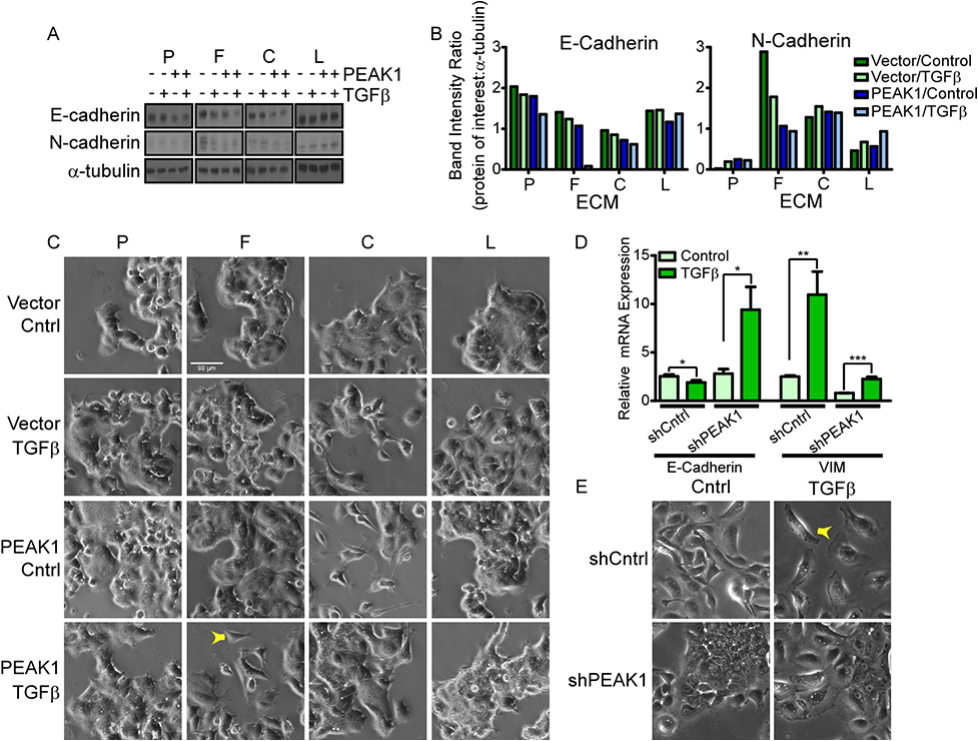
PEAK1 potentiates fibronectin/TGFβ-induced EMT-like responses. (A-C) MCF7-Vector and-PEAK1 cells were plated on 3ug/mL Fibronectin (F), Collagen (C) or Laminin (L), or plated on Plastic (P) and treated chronically with TGFβ. (A) Western blot analysis for E-cadherin, N-cadherin, and α-tubulin was performed after two weeks in culture. (B) Western blot band quantification from (A) using densitometry analysis. (C) Micrographs of cells after two weeks in culture. Yellow arrowhead points to a typical cell that is not packed tightly into epithelial colonies and is mesenchymal. (D & E) qPCR for E-Cadherin and Vimentin expression (D) and micrographs (E) from CA1h-shCntrl and-shPEAK1 cells plated on fibronectin and treated with TGFβ or vehicle control for 48 hours. (Scale bar: 90μm). Yellow arrowhead points to a typical cell that is not packed tightly into epithelial colonies and is mesenchymal.

We next asked whether PEAK1 expression can influence the EMT response in breast cancer cells when exposed to TGFβ. MCF7-Vector and-PEAK1 cells were plated on plastic or different ECM protein substrates and then chronically treated with TGFβ or vehicle prior to collecting lysates. As shown in Fig 2A and 2B, PEAK1 overexpression in combination with chronic TGFβ treatment had synergistic effects on E-cadherin downregulation only when cells were plated on a fibronectin substrate. While both TGFβ treatment or PEAK1 overexpression could downregulate E-cadherin on other substrates, there was no further additive effect for TGFβ treatment of MCF7-PEAK1 cells under these other substrate conditions (Fig 2A and 2B). In contrast, N-cadherin levels did not change appreciably across ECM substrates in the presence of PEAK1 overexpression and/or TGFβ treatment, confirming the minimally invasive nature of MCF7 cells (Fig 2A and 2B). We also evaluated cell morphology of these same cells under the same conditions in sub-confluent cultures. While either TGFβ treatment or PEAK1 overexpression was able to shift the cell morphology across substrate conditions, an additive increase in mesenchymal morphology (i.e., reduced cell-cell packing and increased spreading or pseudopod formation) was only observed in MCF7-PEAK1 cells treated with TGFβ when they were plated on fibronectin (Fig 2C and S6A Fig). Finally, we evaluated gene expression and morphology changes of the CA1h cells containing either control or PEAK1-specific shRNAs when plated on fibronectin and cultured in the presence or absence of TGFβ. In agreement with the above data from the MCF7 cell variants, TGFβ reduced E-Cadherin and increased Vimentin levels in the CA1h-shCntrl cells indicating EMT induction (Fig 2D). This corresponded to a striking shift toward a mesenchymal-like morphology in the cells (Fig 2E). Specifically, these cells spread more, lose cell-cell contacts, have significant membrane ruffling and acquire a more spindled shape. Notably, silencing PEAK1 alone caused Vimentin levels to decrease (Fig 2D) and the CA1h cells to adopted an epithelial and tightly packed morphology (Fig 2E). Furthermore, PEAK1 knockdown enabled TGFβ to induce E-Cadherin levels (Fig 2D) and a more epithelial morphology (Fig 2E)—thus, PEAK1 is necessary for TGFβ-induced EMT in these cells.

### PEAK1 is necessary and sufficient for increased breast cancer cell migration in response to TGFβ/fibronectin treatment

**Fig 3:**
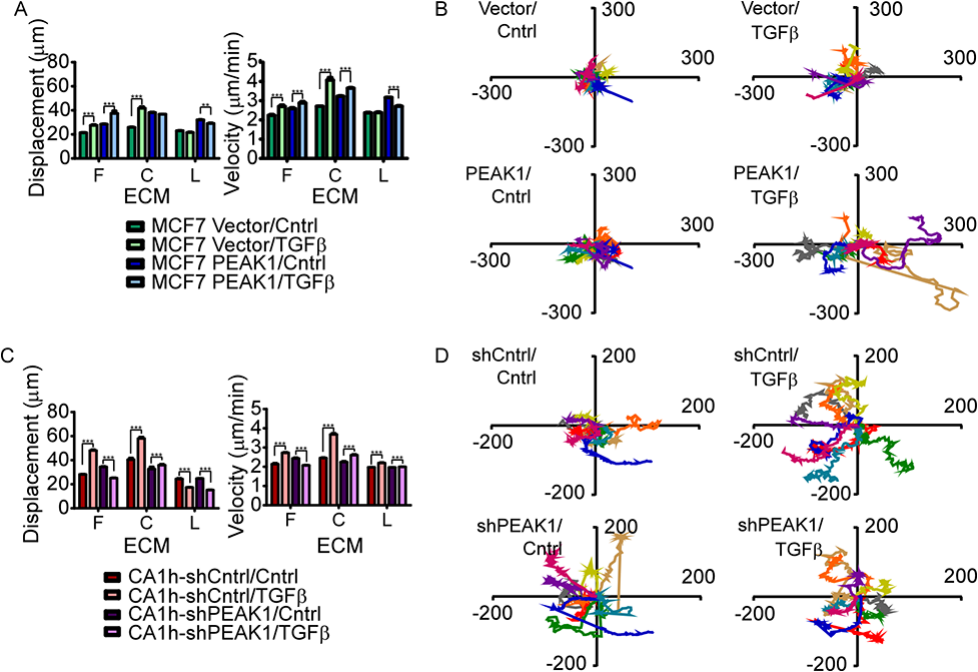
PEAK1 is necessary and sufficient for increased breast cancer cell migration in response to TGFβ/fibronectin treatment. (A-D) MCF7-Vector and–PEAK1 or CA1h-shCntrl and–shPEAK1 cells were chronically treated with TGFβ while being cultured on plastic. (A and C) Single cell migration assays were performed on these cells after plating on the indicated substrates with 3 images per condition being collected every 10 minutes for 24 hours. Cells were tracked using Fiji software to determine Displacement (μm) (left y-axis) and Velocity (μm/min) (right y-axis). Two-way ANOVA statistical analysis was performed using Prism. (B and D) 10 representative cell tracks for each of the indicated cell populations are shown when cells were migrating on fibronectin. *** indicate p-values < 0.001.

Since cell migration is a behavior that traditionally increases as breast cancer cells acquire more mesenchymal and invasive character [35], we next tested the function of PEAK1 in regulating breast cancer cell migration velocity, displacement and migration track length following TGFβ treatment of either MCF7 and CA1h cells plated on different ECMs. As shown in Fig 3A, PEAK1 overexpression in combination with TGFβ treatment, displayed an additive effect on both cell velocity and displacement only when the cells migrated on fibronectin. In agreement with these data, the track lengths of MCF7-PEAK1 cells on fibronectin after TGFβ treatment were significantly longer than those of either PEAK1 overexpressing or TGFβ treated cells alone (Fig 3B). In contrast, chronic treatment of control CA1h cells with TGFβ stimulated their migration significantly on all substrates, while PEAK1 knockdown in these cells reversed this effect of TGFβ in the context of fibronectin only (Fig 3C and 3D). Taken together, these results clearly indicate that TGFβ-induced motility in breast cancer cells is potentiated by fibronectin in a PEAK1-dependent manner.

### PEAK1 cooperates with fibronectin to block the cytostatic effects of TGFβ

**Fig 4:**
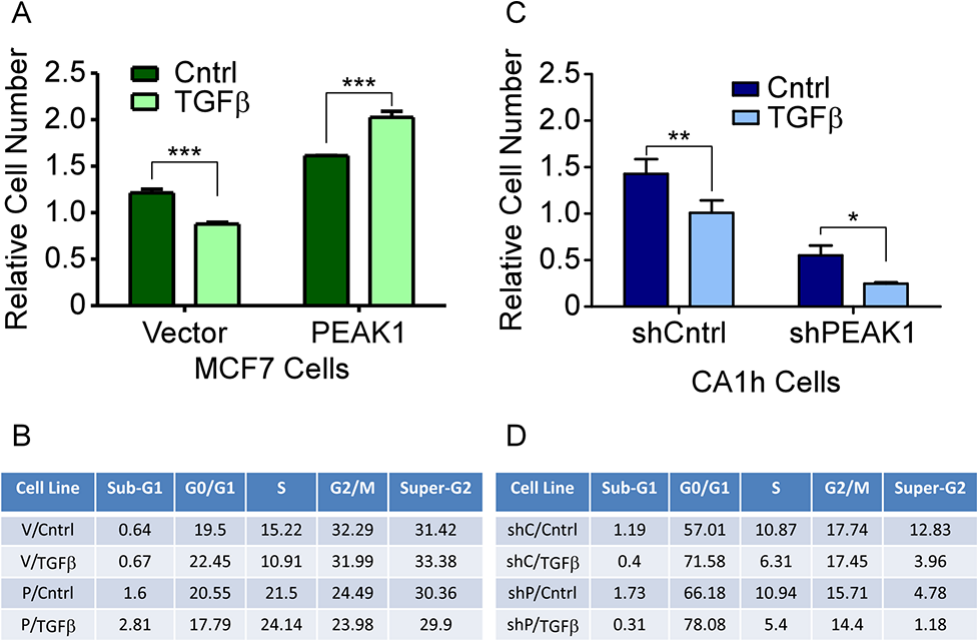
PEAK1 cooperates with fibronectin to block the cytostatic effects of TGFβ. MCF7-Vector and–PEAK1 or CA1h-shCntrl and–shPEAK1 cells were plated on fibronectin then treated with TGFβ. (A and C) After 72 hour incubation, an AQueous One assay was performed. The relative cell number was plotted and two-way ANOVA analysis was performed to determine statistical significance. (B and D) The cell cycle profiles were analyzed and the percent of cells in each stage are shown. *, **, *** indicate p-values < 0.05, 0.01 and 0.001, respectively.

The conversion of TGFβ signaling to a pro-EMT factor in cancer often correlates with its inability to inhibit cell proliferation [36,37]. Therefore, we evaluated the number of viable cells as well as the cell cycle profiles for the MCF7 and CA1h cell lines in response to TGFβ when plated on fibronectin. While TGFβ treatment of the MCF7-Vector cells caused a significant decrease in viable cell number and S-phase percentages, MCF7-PEAK1 cells responded to TGFβ treatment with increased viable cell numbers and S-phase percentages (Fig 4A and 4B). As has been previously reported [36,37], we observed that TGFβ treatment of CA1h-Cntrl cells reduced cell proliferation (Fig 4C and 4D). Still, silencing PEAK1 alone decreased the number of viable CA1h cells and S-phase percentages while increasing the G0/G1 percentages (Fig 4C and 4D). Furthermore, TGFβ treatment of the CA1h-shPEAK1 cells almost completely blocked cell proliferation (Fig 4C and 4D). Importantly, the decrease in proliferation was not likely due to an increase in apoptotic-like cells since the sub-G0/G1 percentages remained unchanged (Fig 4D), indicating that the combined effect of PEAK1 knockdown and TGFβ treatment on fibronectin is due to cellular senescence.

### PEAK1 is required for TGFβ/fibronectin-induced metastasis in vivo

**Fig 5:**
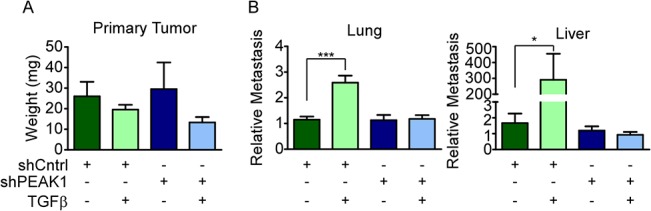
PEAK1 is required for TGFβ/fibronectin-mediated metastasis *in vivo*. (A and B) CA1h-shCntrl and–shPEAK1 cells were plated on fibronectin then treated with TGFβ for 72 hours, after which a CAM assay was preformed. (A) After harvesting the primary tumor, it was weighed. (B) qPCR for chicken GAPDH and human *alu* was performed on genomic DNA extracted from the liver and lung tissue. A t-test was performed to determine statistical significance. *, **, *** indicate p-values < 0.05, 0.01 and 0.001, respectively.

The combined effect of TGFβ and fibronectin on the metastatic potential of MCF10CA1h cells *in vivo* has not been previously evaluated. Based upon the cell morphology data in Fig 2, we predicted that TGFβ treatment of these cells in the presence of fibronectin would induce a more invasive and metastatic response. To test this, we used the chorioallantoic membrane (CAM) of the developing chicken (*Gallus gallus*) as an *in vivo* xenograft model for growth, invasion and metastasis of cancer cells [38]. Briefly, the primary tumor weight from the CAM was measured and qPCR was used to measure the amount of human *alu* repeat sequences in both lung and liver tissue seven days after xenografting. As has be previously reported [38], the levels of human *alu* repeat sequences in these respective tissues can be used as an indicator of relative metastatic potential. PEAK1 silencing and/or TGFβ treatment in the CA1h cells grown on fribronectin prior to xenografting did not affect tumor growth on the CAM (Fig 5A). Notably, however, TGFβ treatment of the CA1h-shCntrl cells strongly induced metastasis of these cells to both lung and liver tissue. However, PEAK1 knockdown completely abrogated this effect (Fig 5B). Importantly, these data demonstrate for the first time that the cooperative effects of TGFβ and fibronectin on breast cancer metastasis require PEAK1 kinase expression/function. Furthermore, the fact that PEAK1 knockdown alone does not abrogate the metastatic potential of these cells demonstrates a specific role for PEAK1 in TGFβ-induced metastasis in breast cancer.

### PEAK1 potentiates TGFβ-induced Src/MAPK signaling in a fibronectin-dependent manner

**Fig 6:**
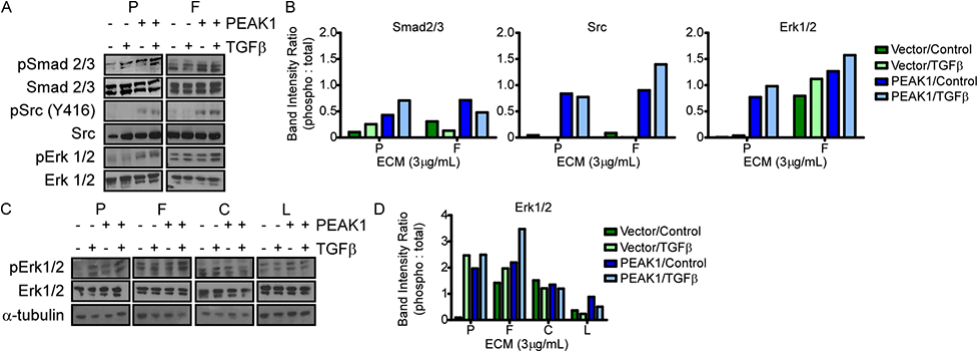
PEAK1 potentiates TGFβ-induced Src/MAPK signaling in a fibronectin-dependent manner. (A and C) MCF7-Vector and-PEAK1 cells were plated on the indicated substrates (i.e., Fibronectin—F, Collagen—C, Laminin—L, or Plastic–P). Cells were then serum starved for 8 hours, then stimulated with TGFβ for 30 minutes. Lysates were collected and Western blot analysis was done for indicated proteins and phospho-proteins. (B and D) Band intensity ratio for phospho-protein to total protein was calculated for western blots from A and C, respectively.

Next, we evaluated the effect of PEAK1 overexpression on TGFβ-induced Smad2/3, Src and MAPK signaling in MCF7 breast cancer cells. On plastic, PEAK1 overexpression alone increased activation of Src, Erk1/2 and Smad2 pathways, but also acted in a cooperative and additive fashion with short-term TGFβ stimulation to activate Smad2/3 to a greater extent (Fig 6A and 6B). Plating the MCF7-Vector cells on fibronectin alone robustly stimulated activation of the Erk1/2 pathway (Fig 6A and 6B). PEAK1 overexpression in the context of fibronectin led to a robust activation of Src and a modest increase in both Erk1/2 and Smad2/3 signaling over fibronectin alone (Fig 6A and 6B). Notably, however, TGFβ treatment of either MCF7-Vector or-PEAK1 cells on fibronectin was unable to further activate Smad2/3, but preferentially increased Src and Erk1/2 signaling. Importantly, this effect of TGFβ switching from Smad2/3 to Src-Erk1/2 signaling pathways in the context of fibronection was potentiated by PEAK1 overexpression (Fig 6A and 6B). In order to determine if this effect was specific to fibronectin, we looked at Erk1/2 activity under these same conditions in both MCF7-Vector and-PEAK1 cells when cultured on plastic or different ECM protein substrates. Not only did we observe the highest magnitude of Erk1/2 signaling in PEAK1 overexpressing MCF7 cells treated with fibronectin and TGFβ, but neither plastic, collagen nor laminin were able to cooperate with TGFβ to induce PEAK1-dependent Erk1/2 activation (Fig 6C and 6D).

### PEAK1 and TGFβ Cooperate to Promote Resistance to Src Kinase Inhibition

**Fig 7:**
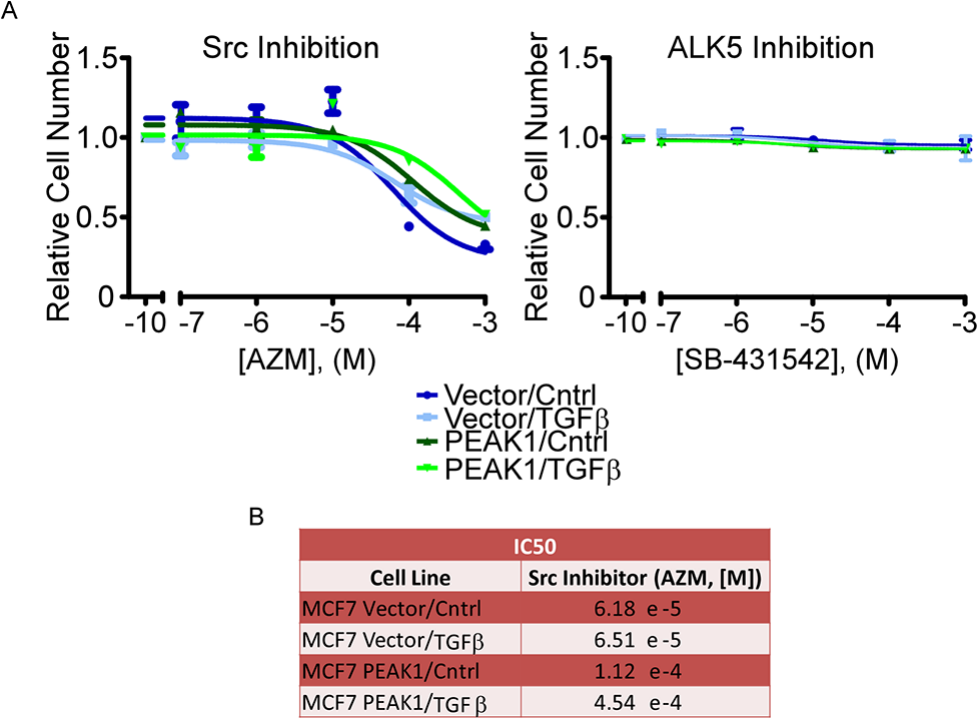
PEAK1 and TGFβ cooperate to promote resistance to Src kinase inhibition. (A) Indicated populations of MCF7-Vector and–PEAK1 cells were plated on fibronectin and treated with increasing doses of either AZM (left) or SB-431542 (right). After 72 hours an AQueous One assay was performed to assess cell viability. Relative cell number versus drug concentration (M) is plotted. (B) AZM IC_50_ values for are reported for the indicated cell population.

Our previous work has shown that PEAK1 promotes therapy resistance in human cancers [18,39], and others have reported that EMT induction can also drive therapy resistance [21]. Therefore, we assessed the cooperative effect of TGFβ treatment and PEAK1 overexpression on Src (AZM) and ALK5 (SB-431542) kinase inhibition responses–two previously identified therapeutic targets in human malignancies [40,41]. Interestingly, ALK5 inhibition did not decrease cell viability in any of the cell variants, even at high doses, suggesting that canonical TGFβ signaling is not responsible for cell viability and/or proliferation in these cells (Fig 7A). However, we discovered that PEAK1 overexpression and TGFβ treatment cooperate to reduce the potency of Src inhibition in the presence of fibronectin. Specifically, TGFβ treatment of MCF7-PEAK1 cells on fibronectin increased the AZM IC_50_ value by nearly 10-fold (Fig 7A and 7B).

### PEAK1 regulates TGFβ switching between tumor suppressor and pro-metastatic functions in breast cancer

**Fig 8:**
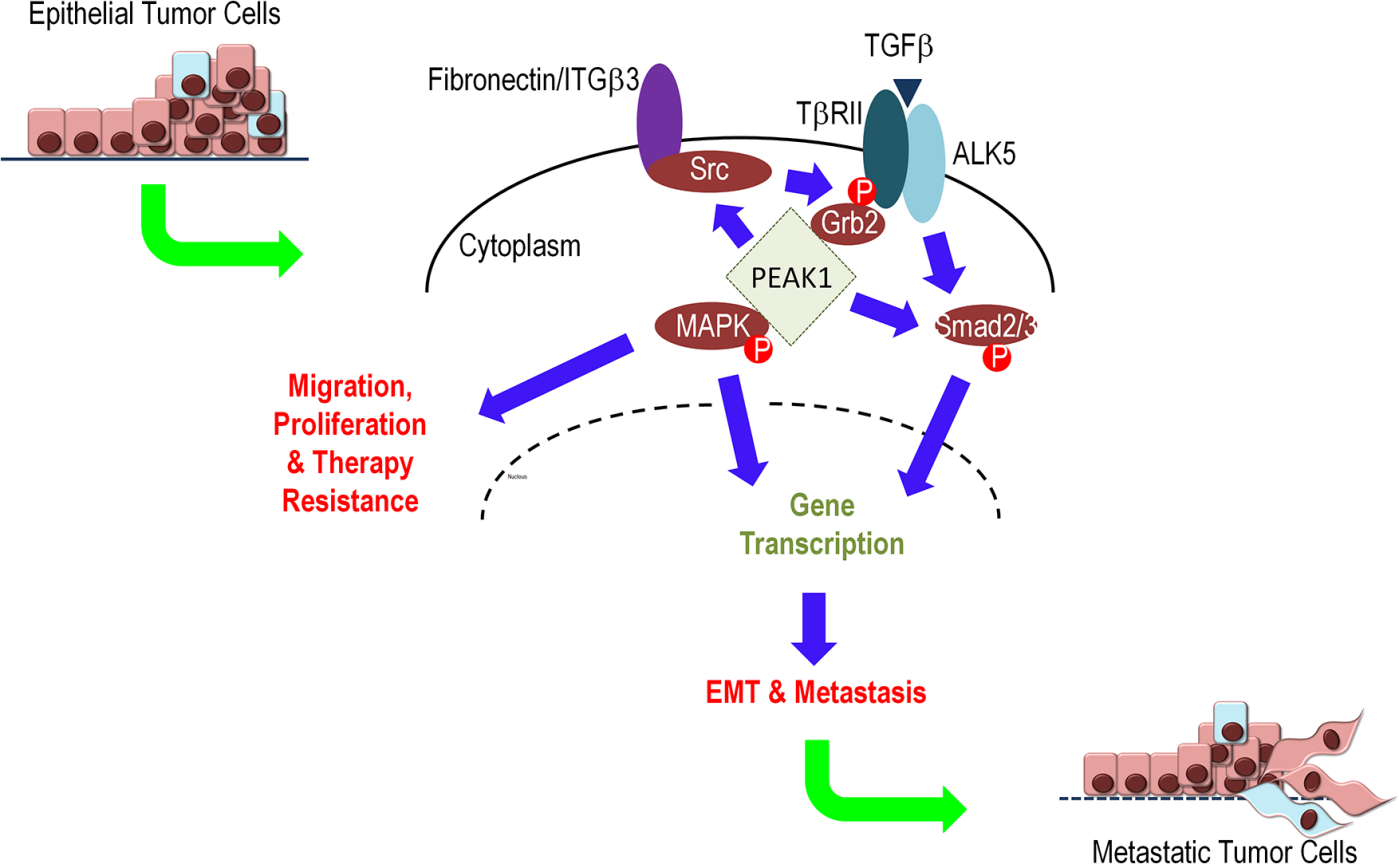
PEAK1 regulates TGFβ switching between tumor suppressor and pro-metastatic functions in breast cancer. PEAK1 can bind and facilitate the recruitment of Src kinase to integrins and TβRII/Grb2 complexes to facilitate non-canonical TGFβ-induced MAPK signaling in the presence of extracellular matrix proteins that signal through ITGB3. Additionally, PEAK1 can promote Smad2/3 in the presence of fibronectin while potentiating TGFβ-induced Smad2/3 signaling in the absence of ECM protein. Ultimately, PEAK1 converts TGFβ signaling from an anti-proliferative growth factor to a pro-tumorigenic one.

Based upon our data and the work of others, we propose a model in which increasing levels of PEAK1 expression can promote the switching of TGFβ responses from anti-proliferative or pro-apoptotic to pro-tumorigenic and pro-metastatic. Importantly, as many others have noted, TGFβ responses depend upon context, and our data demonstrate that PEAK1-mediated switching of TGFβ signaling and function occurs in the context of fibronectin/integrin signaling. More specifically, when PEAK1 is upregulated in the presence of fibronectin, TGFβ signaling can co-regulate Smad2/3 and MAPK signaling to promote EMT, tumor cell migration/proliferation and cancer metastasis (Fig 8).

## Discussion

TGFβ is a pleiotropic growth factor that elicits its cellular responses in a context-dependent fashion via both canonical and non-canonical signaling pathways [8,9]. While significant efforts have been put forth to understand the molecular details that underscore these varied responses [9,42,43], the precise mechanisms that regulate TGFβ signaling in various contexts remain to be fully elucidated. In human cancers, TGFβ can promote growth arrest and apoptosis in pre-malignant tissues, while facilitating metastasis through EMT and the acquisition of invasive behavior in later stages [7]. As such, determining the context in which TGFβ antagonists can improve disease outcome for cancer patients is of great interest within the cancer research community [44].

We report here that PEAK1 kinase is a novel molecular regulator of TGFβ signaling responses, and plays a critical role in determining the context in which TGFβ signaling elicits tumor suppressive or pro-tumorigenic functions in breast cancer cells. Furthermore, we also report that PEAK1 expression levels significantly and positively correlate with EMT gene expression signatures in patient samples and ER- and/or HER2-positive breast cancer cell populations (S1 Fig). Thus, PEAK1 levels or a PEAK1 gene expression signature may be important indicators for determining when TGFβ blockade is a viable anti-cancer treatment option. While further studies will be essential to test this hypothesis, several lines of evidence exist to support this possibility. First, our work (Figs 1C–1E, 2, 3 and 5 and S6 Fig) and the work of others [45] have demonstrated that TGFβ induces EMT/migration/invasion/metastasis in breast cancer cells with high PEAK1 expression (i.e., MCF10CA1h and MDA-MB-231), but has little or no effect on migration/invasion in low PEAK1-expressing breast cancer cells (i.e., MCF-7 and MCF10CA1a). Second, previous work has demonstrated that the SPARC gene is a highly upregulated mesenchymal gene following PEAK1-induced EMT in mammary epithelial cells [21]. In parallel, SPARC has been reported to regulate TGFβ’s ability to promote mesenchymal cell behavior [46,47]. Thus, identifying tumor subtypes in which PEAK1 levels or PEAK1-induced EMT gene signatures are elevated may facilitate the selection of patients that will respond positively to anti-TGFβ treatment regimens.

In addition to functioning as a biomarker, our results suggest that PEAK1 may be a viable target for reversing the cellular response to TGFβ action in more advanced stages of breast cancer. Specifically, we demonstrate that PEAK1 overexpression converts TGFβ to a pro-proliferative factor in MCF7 cells (Fig 4A and 4B), and that PEAK1 knockdown cooperates with TGFβ to almost completely block proliferation in MCF10CA1h cells (Fig 4C and 4D). Notably, this switching of TGFβ to a pro-proliferative factor in the presence of high PEAK1 levels correlates with PEAK1’s ability to promote TGFβ-induced motility, EMT gene expression, mesenchymal cell morphology and tumor metastasis (Figs 2, 3, 4 and 5 and S6 Fig). Thus, by targeting PEAK1 protein-protein interactions or the regulators of PEAK1 expression breast cancer cell growth can be significantly reduced and/or cells can be made more sensitive to chemo- or targeted-therapies. The viability of this approach is supported by our previous work demonstrating that PEAK1 promotes the *in vivo* growth of both breast and pancreatic cancer cells [17,18]. Additionally, we’ve reported that the translation factor eIF5A drives PEAK1 protein production and can be targeted with the FDA-approved anti-fungal agent Cyclopirox Olamine (CPX) to both decrease cellular PEAK1 levels and abrogate pancreatic cancer cell growth [39]. While further experimentation is needed to clarify whether CPX-mediated PEAK1 suppression promotes cytotoxicity and/or chemotherapy sensitization by converting the endogenous tone of TGFβ signaling from pro-tumorigenic to anti-proliferative, it is a promising prospect to already have identified a targeted therapeutic (i.e., CPX) that can block PEAK1 function but which shows limited off-target toxicity in clinical trials [48].

Although classified as a pseudokinase [49,50], our previous work [17] has demonstrated that the kinase domain within PEAK1 (aa. 1330–1664) has tyrosine kinase activity. Furthermore, we’ve shown that the PEAK1 double point-mutant (K1369M/D1516A), which is predicted to lack ATP-dependent activity, is unable to promote the proliferation of human pancreatic epithelial cells further suggesting a role for the catalytic functions of PEAK1 in its pro-tumorigenic roles [18]. While it is possible that the kinase domain plays an important role in PEAK1-mediated TGFβ switching, further studies are needed to evaluate the efficacy of PEAK1 kinase activity as well as potential substrates in this context. However, previous work from our group [17,18] and that of others [19,21,51], which shows that PEAK1 interacts with Src, Grb2 and Shc1 and can activate MAPK signaling pathways, suggests a scaffolding mechanism by which PEAK1 drives TGFβ-induced tumorigenic behavior. Work from the Schiemann laboratory has characterized novel mechanisms by which TGFβ converts from being a Smad2/3-dependent tumor suppressor to a Smad2/3-independent tumor promoter. More specifically, they have shown that extracellular matrix fibronectin (or other ECMs that bind/activate integrin beta 3) recruits Src kinase to phosphorylate TβRII at tyrosine 284. This leads to Grb2-TβRII binding and downstream MAPK signaling that can drive tumor progression in models of breast cancer [11,12]. These previous studies together with our current work demonstrating that PEAK1 promotes TGFβ-induced MAPK signaling in the presence of extracellular fibronectin (Fig 6) places PEAK1 at the center of this signaling cascade (Fig 8). While further work is necessary to elucidate the cellular biochemistry of these signaling events and the molecular determinants within PEAK1 that are essential to facilitate TGFβ-induced MAPK activation in this context, it is useful to consider how antagonists of PEAK1 (e.g., CPX) can be leveraged in combination with existing therapeutic interventions in breast cancer (e.g., Src kinase inhibition) to combat this class of malignancies. In this regard, future studies should evaluate the success of such targeting combinations using preclinical *in vitro* and *in vivo* tumor models.

In summary, PEAK1 mediates the switch in TGFβ signaling from tumor suppressor to tumor promoter in the context of extracellular fibronectin. Furthermore, PEAK1 is necessary and sufficient for TGFβ-induced/fibronectin-dependent metastasis and resistance to Src kinase inhibition. This further understanding of the role of PEAK1 in TGFβ signaling during breast cancer progression should pave the way for the development of targeted therapies to block breast cancer progression and increase patient survival.

## Methods

### Cell Culture

MCF7 and MDA-MB-231 cells were obtained from the American Tissue Culture Collection (ATCC). MCF7 cells were cultured in DMEM/High-Glucose growth media supplemented with 10% FBS, and 10μg/mL insulin. MDA-MB-231 cells were cultured in DMEM/High-Glucose growth media supplemented with 10% FBS. MCF7 Vector and MCF7 PEAK1 were generated from the parental line in Dr. Richard Klemke’s laboratory as previously published [17]. MCF10A, MCF10AT1k, MCF10CA1h and MCF10CA1a cells were purchased from the Karmanos Cancer Center (made in the laboratory of Dr. Fred Miller). MCF10A cells were cultured in DMEF12 growth media, supplemented with 5% horse serum, 10μg/mL insulin, 20ng/mL EGF, 0.5μg/mL hydrocortisone, 100ng/mL cholera toxin and antibiotics. MCF10AT1K cells were cultured in DMEF12 growth media, supplemented with 5% horse serum, 10μg/mL insulin, 20ng/mL EGF, 0.5μg/mL hydrocortisone and antibiotics. MCF10CA1h and MCF10CA1a cells were cultured in DMEF12 growth media and supplemented with 5% horse serum and antibiotics.

### TGFβ Treatments

Acute—Cells were plated at 3x10^5^ cells/mL in 2mL and left to grow to 70% confluent. Cells were then serum starved for 8 hours, treated for 30 minutes with 2.5ng/mL TGFβ of 0.1% BSA. Cells were lysed with RIPA Lysis Buffer containing phosphotase and protease inhibitors and a western blot was performed. Chronic—Cells were plated at 1x10^5 cells/mL in 2mL in a 6-well plate and left to attach overnight. Cells were then treated with 2.5ng/mL TGFβ or 0.1% BSA. Cells were maintained (media changed every 48 hours and passaged when necessary) and retreated every 48 hours. Cells were expanded as needed.

### Lentiviral Transduction

MCF10CA1h cells were plated at 1.6x10^4^ cells/well into a 96-well plate and left to attach overnight. Cells were then treated with 10μL of viral particles containing a puromycin resistant pKLO.1 vector with a scramble shRNA or PEAK1-specific shRNA (3’-UTR targeting) in 110μL of complete media and left to incubate for 18hrs, after which the media was changed. The following day media was changed and supplemented with 10μg/mL puromycin. Media was changed with puromycin supplemented media every 3 days until resistance was definite. Cells were then maintained in media containing puromycin.

### Single Cell Migration Assays

Wells of a 24-well plated were coated for one hour with 3μg/mL of fibronectin, collagen, or laminin. Cells were plated at 1x10^4^ cells/mL in 1mL of complete media and allowed to attach for 5 hours. After 5hours, wells were filled with media containing 25mM HEPES, sealed and imaged every 10 minutes for 24 hours. TGFβ or 0.1% BSA treatment was maintained throughout plating, sealing and imaging. Plates were imaged using a Leica widefield-brightfield phase contrast microscope and the data was analyzed with Fiji (Image J) software. Two-way ANOVA analysis was performed to determine statistical significance.

### Wound Healing Assays

Wells of a 24-well plated were coated for one hour with 3μg/mL of fibronectin, collagen, or laminin. MCF10 cells were plated at 8x10^5^ cells/mL in 1mL of complete media. MCF7 cells were plated at 4x10^5^ cells/mL in 1mL of complete media. Wells were treated with indicated treatments when necessary. Cells were allowed to attach overnight, then filled with media containing 1M HEPES, sealed and imaged every 10 minutes for 24 hours. Plates were imaged using a Leica widefeild-brightfeild phase contrast microscope and the data was analyzed with Fiji software. Two-way ANOVA analysis was performed to determine statistical significance.

### Proliferation Assays

Wells of a 24-well plated were coated for one hour with 3μg/mL of fibronectin, collagen, or laminin. Cells were plated at 1x10^4^ cells/mL in 200μL and allowed to attach overnight. Wells were then treated with the indicated treatments and imaged at 0, 24, 96 and 168 hours post-treatment. Cells per image were counted and graphed. Plates were imaged using a widefeild-brightfeild phase contrast microscope and the data was analyzed with Fiji software. Two-way ANOVA analysis was performed to determine statistical significance.

### AQueous One Assays

Wells of a 24-well plated were coated for one hour with 3μg/mL of fibronectin, collagen, or laminin. Cells were plated at 1x10^4^ cells/mL in 200μL and allowed to attach overnight. Wells were then treated with the indicated treatments then left for 72 hours. 20μL of AQueous One solution was added to each well and the absorbance was taken at 1.5, 2, and 3 hours after addition of AQueous One Solution. Two-way ANOVA analysis was performed to determine statistical significance.

### Western Blots

Cells were lysed with Stringent RIPA Lysis Buffer and rotated at 4°C for 3 hours. Lysates were then cleared by centrifugation, and the protein concentration was determined via a Bradford Assay. Then 4–12% Bis-Tris gels were ran and transferred to nitrocellulose membranes. The membranes were then probed at 4C overnight with indicated antibodies with the following dilutions: PEAK1 (abcam 1:1000), PEAK1 (santa cruz 1:500), N-cadherin (cell signaling 1:1000), E-cadherin (cell signaling 1:1000), Smad2/3 (cell signaling 1:1000), phospho-Smad2/3 (cell signaling 1:1000), Src (cell signaling 1:1000), phosphor-Src (Y567) (cell signaling 1:1000), Erk1/2 (cell signaling 1:1000), phospho-Erk1/2 (cell signaling 1:1000), beta-actin (PolySci 1:1000). Secondary antibodies were used at a 1:10,000 or 1:25,000 dilution. Band intensities were quantified using Fiji software after image thresh holding. Pixel intensity was collected from boxes of the same size placed around different bands–either and phospho:total protein or protein of interest:housekeeping protein ratios were calculated and plotted.

### Quantitative PCR

RNA was extracted from whole cells using the Thermo Scientific GeneJet RNA purification kit, following the protocol laid out in the kit specifications. RNA concentrations were determined by NanoDrop. The Fermentas Maxima Universal First Strand cDNA kit was used to synthesize cDNA using 100ng of template and both oligo(dT) and random hexamer primers. The protocol was followed and ran through a thermocycler according to the kit specifications. cDNA concentrations were determined by NanoDrop and then diluted to 22.5ng/mL to perform qPCR. Primers were purchased from Integrated DNA technologies and used at a concentration of 10nmol/mL. 8.75μL of nuclease-free water was mixed with 2.5μL of diluted cDNA, 1.25μL of gene-specific primer, and 12.5μL of Thermo Scientific Maxima SYBR Green. Samples were run on an ABI 7300 instrument.

### Cell Cycle Analysis

Cells were plated at 1x10^5^ cells/mL in 2mL in a 6-well plate coated with 3μg/mL ECM/PBS solution. The following day, cells were treated with either with 2.5ng/mL TGFβ or 0.1% BSA). After 72 hours cells were trypsinized, pelleted and resuspended in 1mL of PBS. Then 2.5mL of 100% ethanol was added to each sample and incubated on ice for 15 minutes. The samples were next centrifuged at 1500rpm for 5 minutes to form a pellet. Samples were resuspended in 500μL of 50μg/mL Propidium Iodide (PI) solution and incubated for 40 minutes at 37°C. PBS was added to each sample, and centrifuged at 1500rpm for 5 minutes to obtain a pellet. The pellet was resuspended in 1mL PBS and analyzed by flow cytometry (FlowJo Analysis Software).

### Dose Response with Kinase Inhibitors

MCF7 Vector and PEAK1 control and TGFβ treated cells were plated at 1x10^4^ cells/mL in 200μL in a 96-well plate coated with 3μg/mL of fibronectin. Cells were then treated with 1000μM, 100μM, 10μM, 1μM, or 0.1μM of AZM or SB-431542. After 72hrs an AQueous One assay was performed.

### CAM in vivo Assay Cell Preparation

MCF10CA1h shRNA cells were plated at 6x10^4^ cells/mL in 10mL in a 10cm plate coated with 3ug/mL fibronectin. The following day cells were treated with either 2.5ng/mL TGFβ or 0.1% BSA. After 72 hours cells were trypsinized, counted and pelleted. Cells were then resuspended at 1x10^6^ cells/20μL of matrigel. Cells were then xenografted onto the CAM of the prepared egg.

### CAM in vivo Assay

Chicken eggs were purchased from Meyer Hatchery, and incubated for 10 days at 37°C, 60% humidity. The CAM Assay was preformed according to a previously described protocol [38]. On day 10 post fertilization, the CAM was dropped and the eggs were windowed. A plastic ring was then placed on the CAM and cells were xenografted within the ring. Surgical tape was then placed over the window, and the egg was then placed back into the incubator. After an additional 7 day incubation, the egg was opened and the primary tumor was removed from the CAM. The embryo was then extracted from the egg and sacrificed. The liver and lung tissue was collected and flash frozen then stored at -80°C until processed. Upon processing, the tissue was thawed on ice, weighed and homogenized in digestion solution. Samples were then heated at 57°C for 5 hours. Genomic DNA was then extracted using Thermo Scientific GeneJet Genomic DNA purification kit. DNA concentration was then quantified by NanoDrop. gDNA was then diluted to 22.5ng/mL and qPCR was preformed for human *alu* repeats and chicken GAPDH.

## Supporting Information

S1 Fig (A) IHC from the Human Cancer Atlas of four different patients (the same four patients as in Fig 2) with elevated or low PEAK1 levels for MUC1, E-Cadherin, Entactin, ZO-1, Laminin-1, Syndecan-1, Goosecoid, SNAI2, β-Catenin, COL1A2, and LEF-1. (B) Using published microarray data from a collection of breast cancer cell lines, the various lines were divided by biomarker status and the data were analyzed for PEAK1 expression relative to EMT gene signatures (epithelial or mesenchymal) that had been previously reported to change in response to PEAK1 expression in mammary epithelial cells. Linear regression analysis was performed and the Pearson r-values and associated p-values were calculated for the line of best fit for each data set.
(TIF)

S2 Fig *Top*: Micrographs along the edge of a wounded monolayer of MCF10A, AT1K, CA1h and CA1a cells. Arrow indicate sites of increased lamellopodia formation. *Bottom*: Micrographs of MCF10A, AT1K, CA1h, and CA1a cells at sub-confluence. Arrow indicates a cell that is more spread and mesenchymal, representative of the whole population of CA1h cells (Scale bar: 90μm).
(TIF)

S3 Fig Single cell migration assays were performed on the indicated cell populations when plated on the various ECM substrates (i.e., Fibronectin—F, Collagen—C, Laminin—L, or Plastic–P). 3 images per condition were collected every 10 minutes for 24 hours. Cells were tracked using Fiji software.10 representative cell tracks for each of the indicated cell populations are shown when cells were migrating on fibronectin.
(TIF)

S4 Fig Using microarray data from previously published breast cancer patient data, the correlation between PEAK1 and TGFβ response genes was observed in relation to clinical outcome and biomarker status. Statistical significance was calculated using a unpaired student’s t-test.
(TIF)

S5 Fig (A) Western blot analysis on MCF7-Vector and–PEAK1 cells for indicated proteins and phosphor-proteins. (B) Proliferation assay with the MCF7-Vector and–PEAK1 cells, plated on plastic and imaged at 0, 24, 96, and 168 hours. Cells were counted using Fiji Software and ANOVA statistical analysis was performed. Cell number vs. time in hours is plotted. (C) Cell cycle analysis of MCF7-Vector and–PEAK1 cells showing cell cycle stage percentages in the inlay. (D) AQueous One assay was performed on the CA1h-shCntrl and-shPEAK1 cells 72 hours after plating on plastic. The relative cell number was plotted and ANOVA statistical analysis was performed using Prism software. The inlay shows a western blot of PEAK1 and β-actin confirming knockdown of PEAK1. (E) Cell cycle analysis of CA1h -shCntrl and–shPEAK1 cells showing cell cycle stage percentages in the inlay. *** indicate p-values < 0.001.
(TIF)

S6 Fig Phase and overlay of GFP channels for MCF7-Vector/Control,-Vector/TGFβ,-PEAK1/Control and–PEAK1/TGFβ cells plated on fibronectin. (TIF)
