# Supplementary figures and images for: PEAK1 Acts as a Molecular Switch to Regulate Context-Dependent TGFβ Responses in Breast Cancer
Source: PLoS One. 2015 Aug 12;10(8):e0135748. doi: 10.1371/journal.pone.0135748 (PMC4533969; doi:10.1371/journal.pone.0135748)

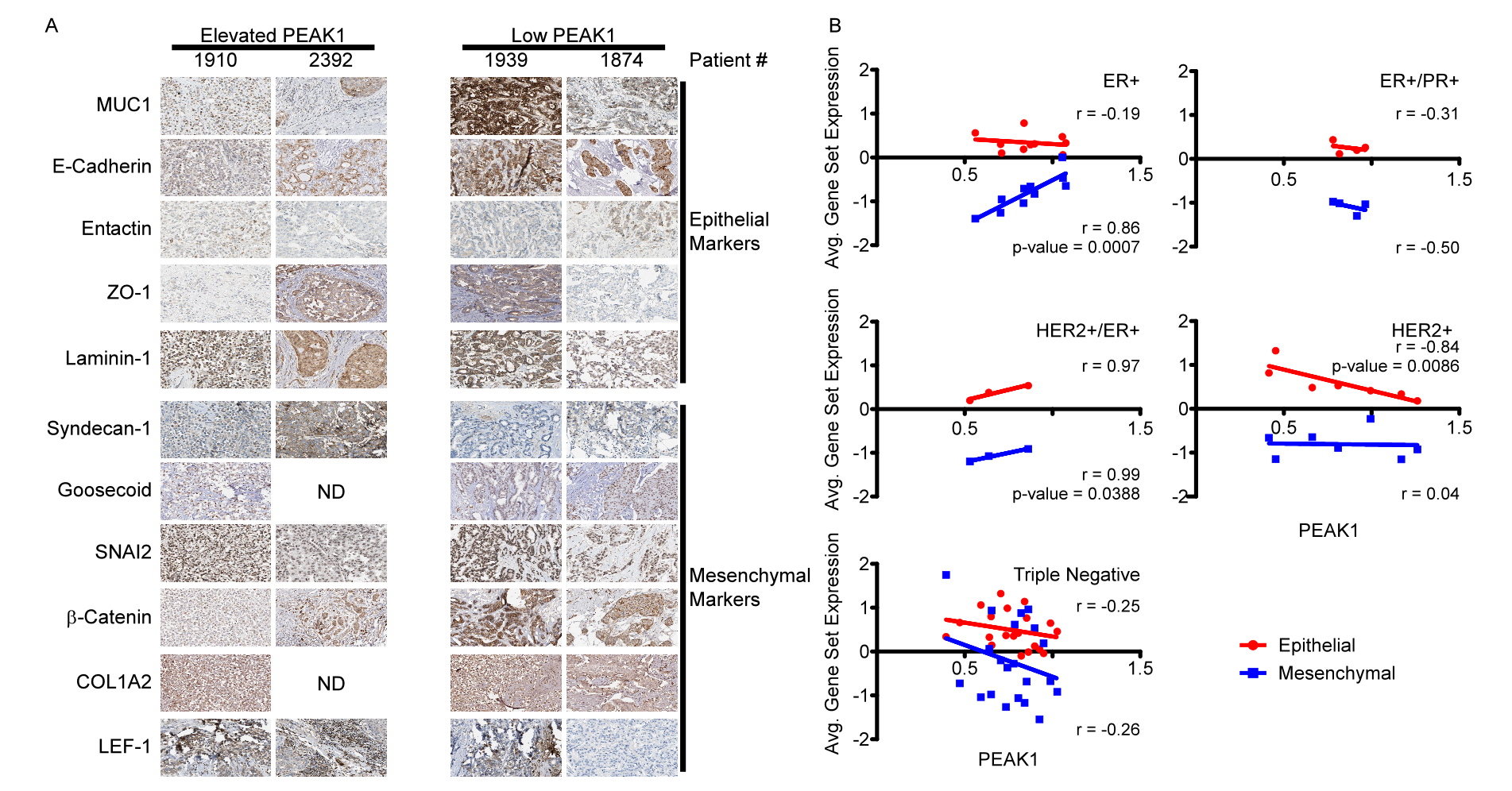

Supplement: S1 Fig — Linear regression analysis was performed and the Pearson r-values and associated p-values were calculated for the line of best fit for each data set. (TIF) [file pone.0135748.s001.tif]

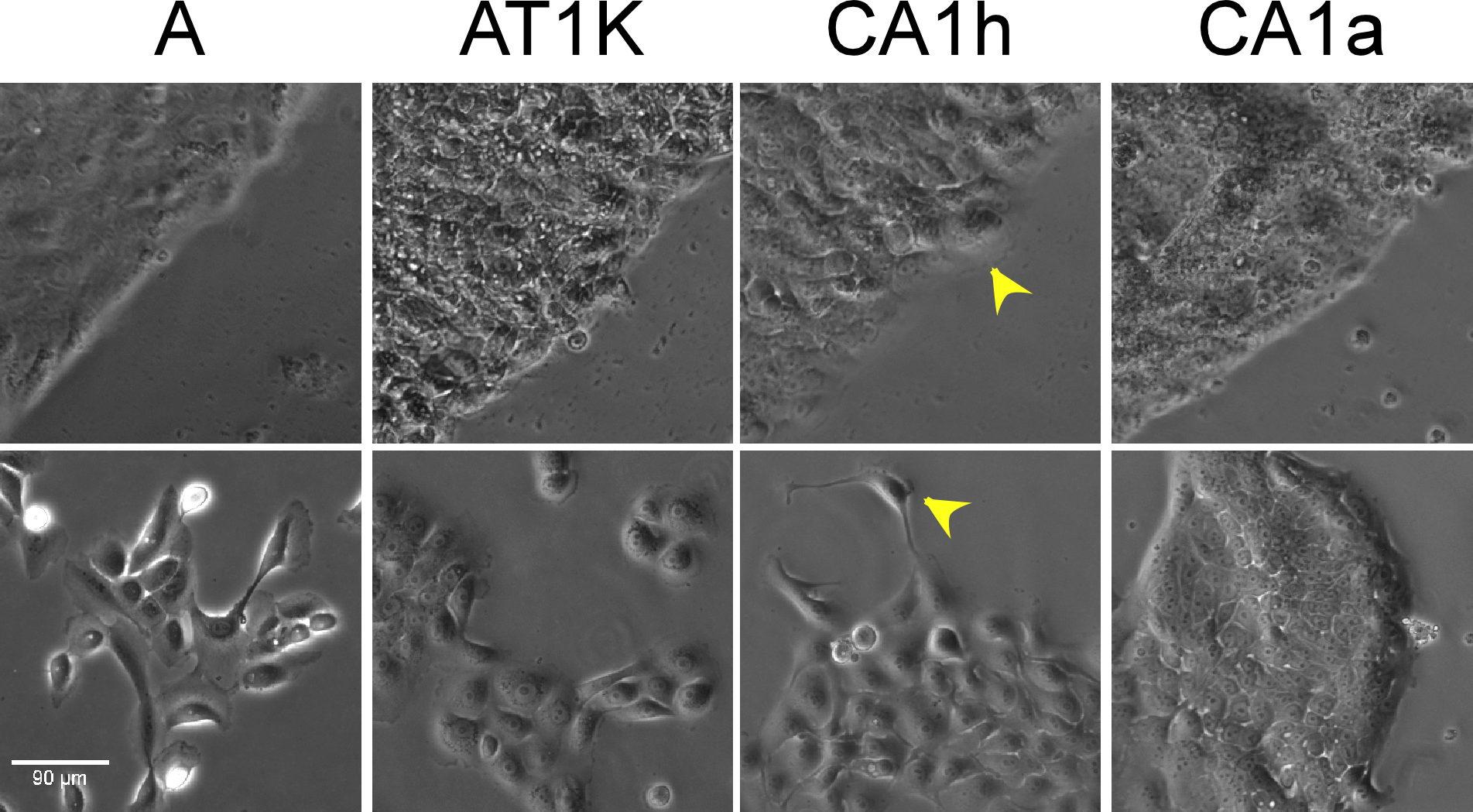

Supplement: S2 Fig — Arrow indicate sites of increased lamellopodia formation. Bottom: Micrographs of MCF10A, AT1K, CA1h, and CA1a cells at sub-confluence. Arrow indicates a cell that is more spread and mesenchymal, representative of the whole population of CA1h cells (Scale bar: 90μm). (TIF) [file pone.0135748.s002.tif]

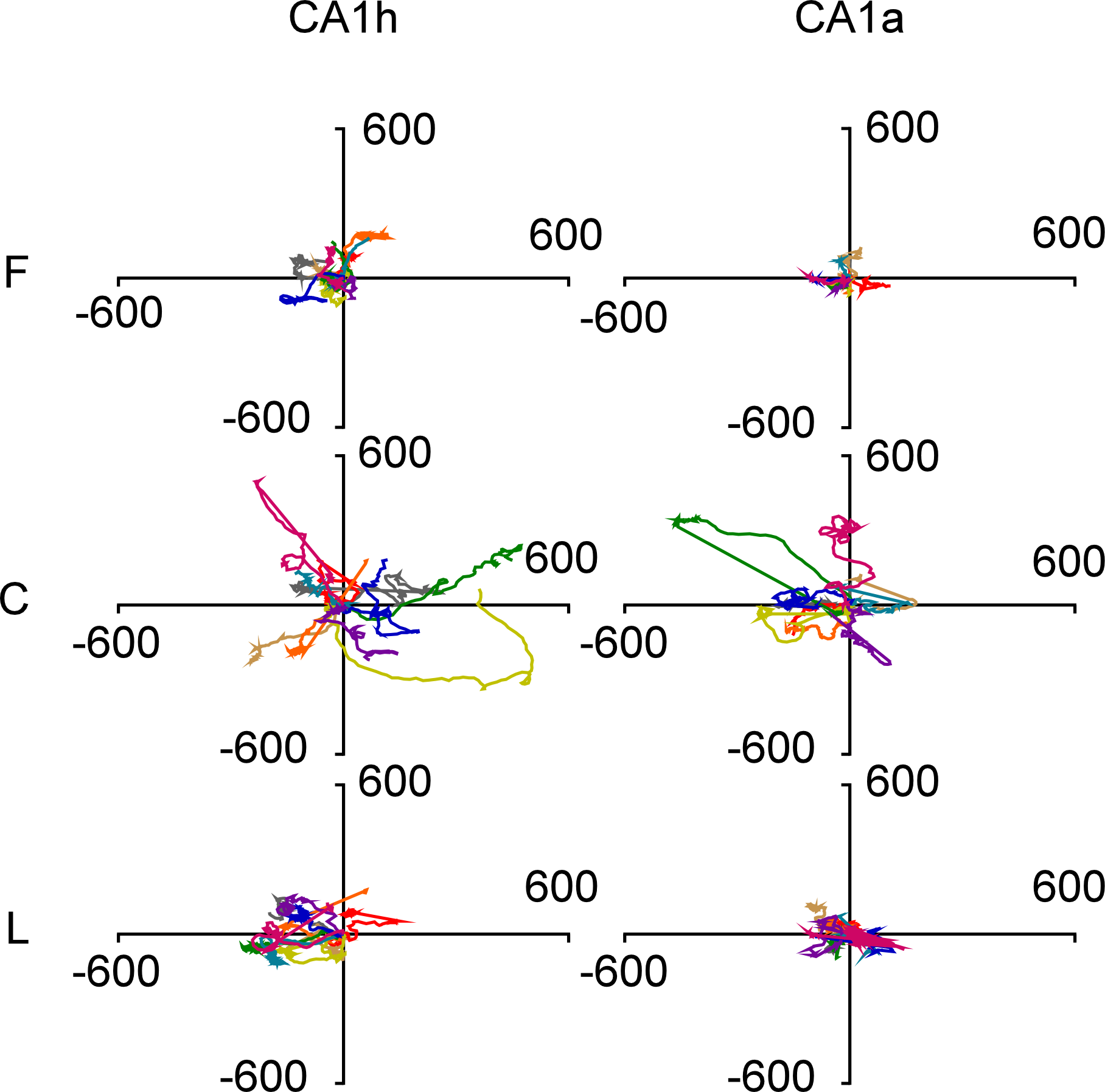

Supplement: S3 Fig — 3 images per condition were collected every 10 minutes for 24 hours. Cells were tracked using Fiji software.10 representative cell tracks for each of the indicated cell populations are shown when cells were migrating on fibronectin. (TIF) [file pone.0135748.s003.tif]

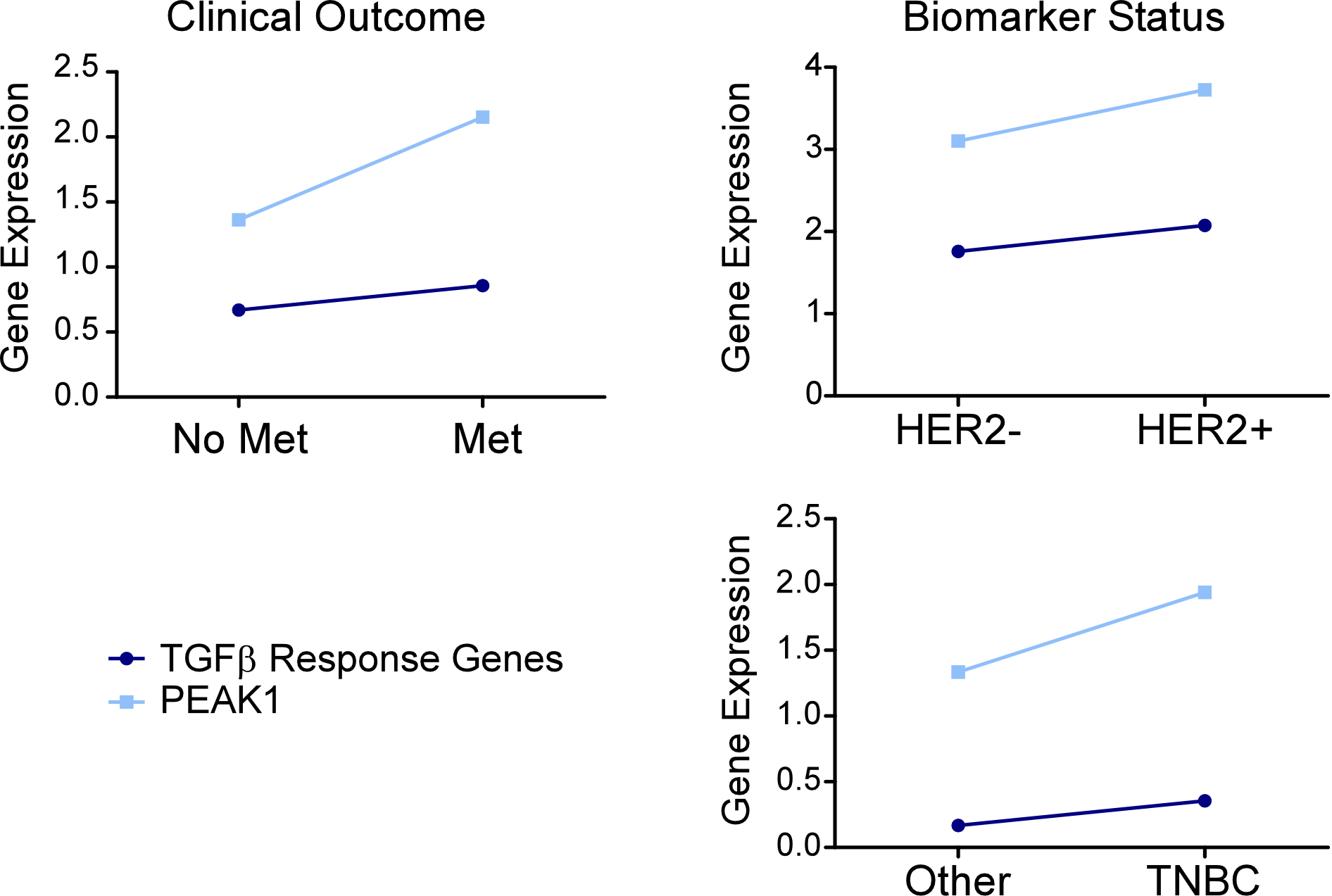

Supplement: S4 Fig — Statistical significance was calculated using a unpaired student’s t-test. (TIF) [file pone.0135748.s004.tif]

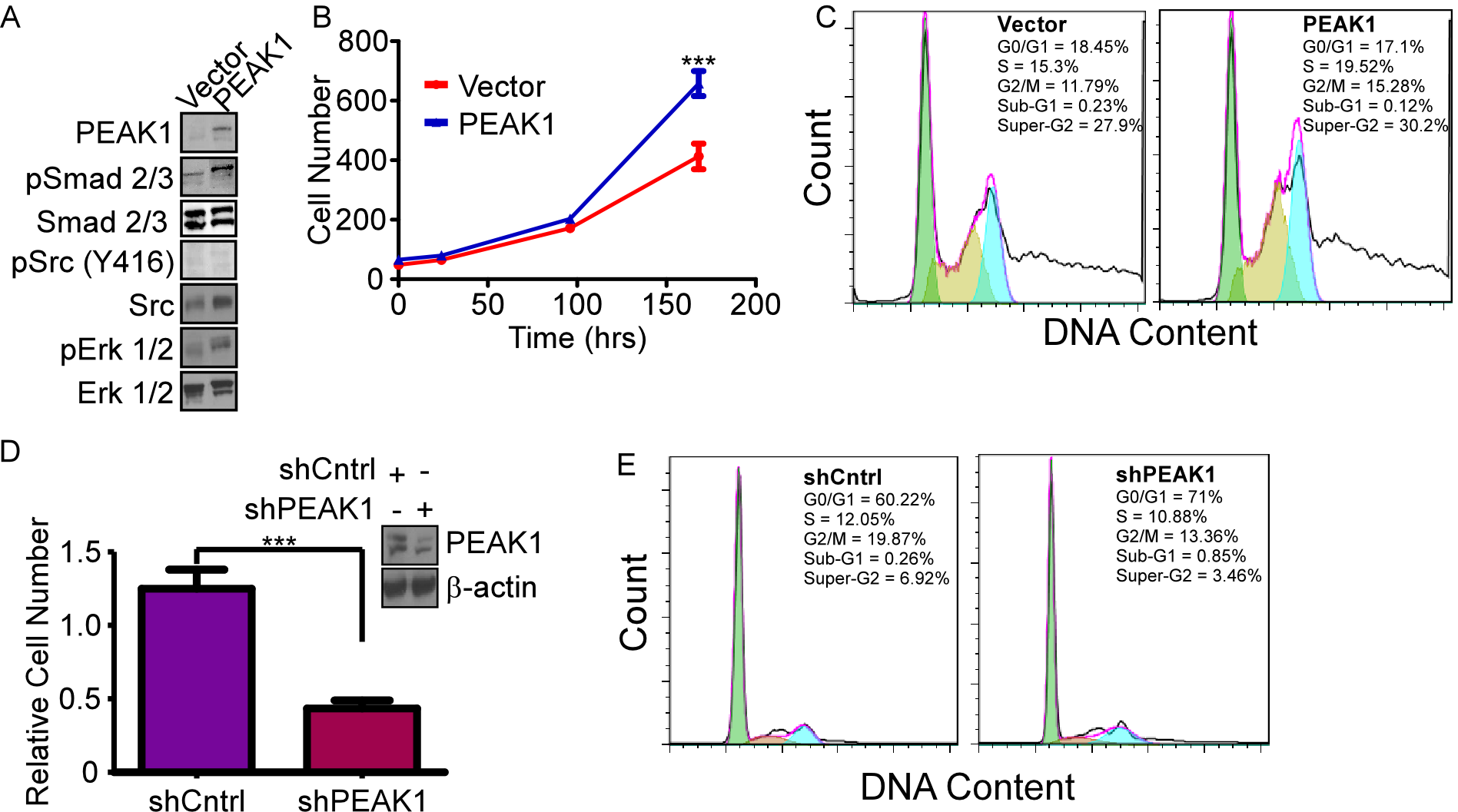

Supplement: S5 Fig — *** indicate p-values < 0.001. (TIF) [file pone.0135748.s005.tif]

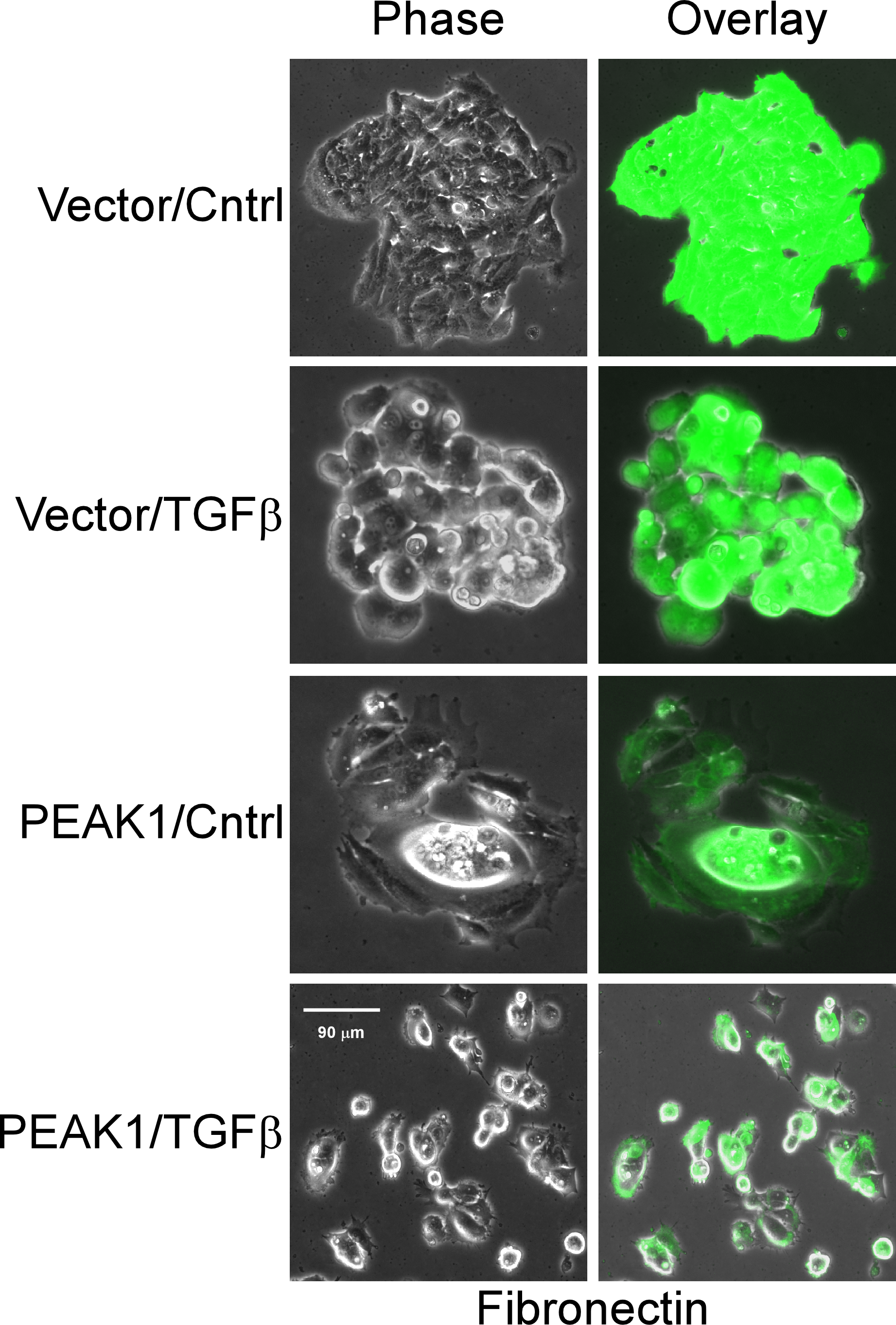

Supplement: S6 Fig — (TIF) [file pone.0135748.s006.tif]
